# A comparative study of the effects of platelet-rich fibrin, concentrated growth factor and platelet-poor plasma on the healing of tooth extraction sockets in rabbits

**DOI:** 10.1186/s12903-022-02126-0

**Published:** 2022-03-23

**Authors:** Siying Li, Hongyi Yang, Qinyu Duan, Hongyu Bao, Aodi Li, Wei Li, Junliang Chen, Yun He

**Affiliations:** 1 Oral and Maxillofacial Reconstruction and Regeneration Laboratory, Southwest Medical University, Luzhou, 646000 China; 2 Department of Oral and Maxillofacial Surgery, The Affiliated Stomatology Hospital of Southwest Medical University, Luzhou, 646000 China

**Keywords:** Tooth extraction, Platelet-rich fibrin, Osteogenesis, Bone resorption, Cone beam computed tomography

## Abstract

**Background:**

Autologous platelet concentrate has been widely used to encourage the regeneration of hard and soft tissues. Up to now, there are three generations of autologous platelet concentrates. Many studies have shown that the three autologous concentrates have different effects, but the specific diversities have not been studied. The purpose of this study was to explore and compare the effects of platelet-rich fibrin, concentrated growth factor and platelet-poor plasma on the healing of tooth extraction sockets in New Zealand rabbits.

**Methods:**

A total of 24 healthy male New Zealand white rabbits aged 8–12 weeks were selected. The experimental animals were randomly divided into four groups: three experimental groups were respectively implanted with PPP, CGF and PRF gel after bilateral mandibular anterior teeth were extracted, and the control group did not implant any material. The alveolar bone of the mandibular anterior region was taken at 2, 4 and 8 weeks after operation. The height and width of the extraction wound were detected by CBCT, the growth of the new bone was observed by HE and Masson staining, and the expression of osteogenic genes was detected by real-time PCR. Data were analyzed using IBM SPSS statistical package 22.0.

**Results:**

The radiological results showed that alveolar bone resorption in all groups gradually increased over time. However, the experimental groups showed lower amounts of bone resorption. The histological results showed that new bone formation was observed in all groups. Over time, the new bone trabeculae of the CGF group became closely aligned while those in the PPP and PRF groups remained scattered. PCR results showed that the expression of BMP-2 and ALP was higher in the experimental groups than the control group.

**Conclusion:**

In conclusion, the application of PRF, CGF and PPP in tooth extraction sockets effectively promoted bone regeneration. CGF showed more effective bone induction and tissue regeneration ability in the long term.

## Background

Alveolar bone after tooth extraction undergoes bone resorption due to lack of dental support and functional stimulation, while bone reconstruction is accompanied by osteoclast resorption and fibrous bone filling [1]. 70–80% of the bone loss occurs in the first 3 months after tooth extraction [2].

The resorption and atrophy of alveolar bone will have an adverse effect on subsequent restoration treatment, especially implant placement. They increase the difficulty of implant operation and are not conducive to the stability of implants. Therefore, methods of alveolar sockets preservation are worthy of further exploration, so as to reduce the resorption of the alveolar ridge at the tooth extraction site, and provide more adequate bone volume and a favorable alveolar ridge shape for subsequent treatment. Numerous studies have been dedicated to evaluating the efficacy of different socket-filling biomaterials. Autogenous and allogeneic bone grafts have been recognized as frequently-used methods for decades, however, several limitations, such as extra site of surgery and prolonged surgery, an uncertain infection rate and limited autologous bone alternatives, have restricted their widespread development [3]. The use of synthetic biomaterials as alternative products has continued to develop subsequently, especially prior to implantation [4, 5]. But most exogenous biomaterials still have some uncertainties in bone mineral binding ability, biodegradability and effective antibacterial ability [6].

In recent years, autologous platelet concentrates, such as platelet-rich fibrin (PRF), concentrated growth factor (CGF) and platelet-poor plasma (PPP) have attracted the attention of researchers due to their ability to promote new bone formation [7] and tissue regeneration [8]. PRF is a second-generation platelet concentrate product which is easy to produce without any biological agents. Many studies have suggested that the use of PRF can stimulate wound healing and promote regeneration of hard and soft tissues through the release of a large amount of leukocyte cytokines and platelets, which interact with the fibrin clot to form a haemostatic plug and to slowly release growth factors, but some scholars are still skeptical about its regenerative effect on bone tissues [9–12]. Moreover, others have shown that there is a lack of standardization in its production and application, and that small differences may lead to variable clinical effects [13]. CGF was introduced by Sacco in 2006 [14]. Unlike PRF, it is centrifuged using special centrifuges, and different centrifugation speeds result in a larger, denser, and more abundant growth factor fibrin matrix [15]. A large number of studies have confirmed the advantages of CGF in bone defect repair [16, 17].

PPP is the supernatant of plasma after centrifugation that contains few platelets. Studies showed that PPP seems to have the ability to facilitate wound healing-associated cell function [18]. In recent years, some in vitro studies have compared the similar effects of PPP and PRP in innervation and muscle repair [19, 20], but there are few studies evaluating the role of PPP in bone tissue, and the results are divergent. Hamdan et al. [21] at the cellular level have shown that the difference of concentration due to the vitro test will lead to a great difference in the results, which is also an important factor limiting the development of PPP.

Alveolar bone is the most active bone tissue in the body. Compared with other parts of bone defects, there will be significant resorption in the tooth extraction without intervention, which puts forward high requirements for the effect of bone induction materials used for tooth extraction. At present, some studies on the application of autologous plasma products in the tooth extraction have no consistent or robust results, and most of their studies are limited with the combined application of other osteogenic induction materials, and there are few independent studies on the effect of plasma products without additives. Further carefully designed and long-term observation cycles and multiple observation levels are needed to explore their respective strengths. Therefore, the aim of this study is to compare the effects of PRF, CGF and PPP on the healing of tooth extraction sockets in rabbits and to compare their long-term effects and influence characteristics of different stages from 2 weeks, 4 weeks and 8 weeks continuous observation. And in order to preliminary explore the mechanism of concrete, we also compare their influence on osteogenesis related genes, aims to provide guidance for clinical choice medicine.

## Materials and methods

### Sample size calculation

The sample size was calculated using PASS 15.0 software (NCSS, LLC, Utah, USA). The statistical design was based on comparing the resorption rates of alveolar bone width at the same time after PRF, PPP, CGF were applied to the tooth extraction sockets. The analysis module One-Way-Analysis of Variance F-Tests in the Means was used. According to the results of the preliminary experiment, the mean resorption rates of alveolar bone width in the PPP, CGF, PRF and control groups was 14%, 13%, 16% and 39% respectively. The standard deviation was set as 12%. Statistical significance was set as α = 0.05, with four groups, statistical power of 0.9, and a group allocation ratio of 1: 1: 1. With these parameters, the sample size needed for the current study was six in each group (n represents the number of tooth extraction sockets) (Table 1).

**Table 1:** One-way analysis of variance F-tests numeric results means: 16 13 14 39

	Average	Total			SD of means	SD	Effect	
Power	n	G	N	K	δm	δ	Size	Alpha
0.9581	5.00	4	20	1.00	10.74	10.00	1.0735	0.0500
0.9298	6.00	4	24	1.00	10.74	12.00	0.8946	0.0500
0.9367	7.00	4	28	1.00	10.74	13.00	0.8258	0.0500
0.9013	8.00	4	32	1.00	10.74	15.00	0.7157	0.0500

### Animals and study design

All of the research protocols used in study were approved by the ethical committee of Southwest Medical University, Luzhou, China (Certificate number 201906-1). A randomized controlled study was conducted in accordance with the ARRIVE guidelines and the Directive 2010/63/EU in Europe [22, 23]. Healthy male New Zealand White rabbits weighing 2.0–2.5 kg (average 2.2 kg) and aged 8–12 weeks each were used in this study. All animals were purchased from the Department of Animal Science Central of Southwest Medical University and were taken good care of by professional laboratory technicians. They were housed in a temperature (22 ± 2 °C) and humidity (55 ± 5%) controlled room under a 12/12 h light/dark cycle and kept in separate cages, with free access to food and water. After 2 weeks of observation, the experimental treatment was carried out.

### Preparation of autologous PPP, CGF, and PRF

**Fig. 1:**
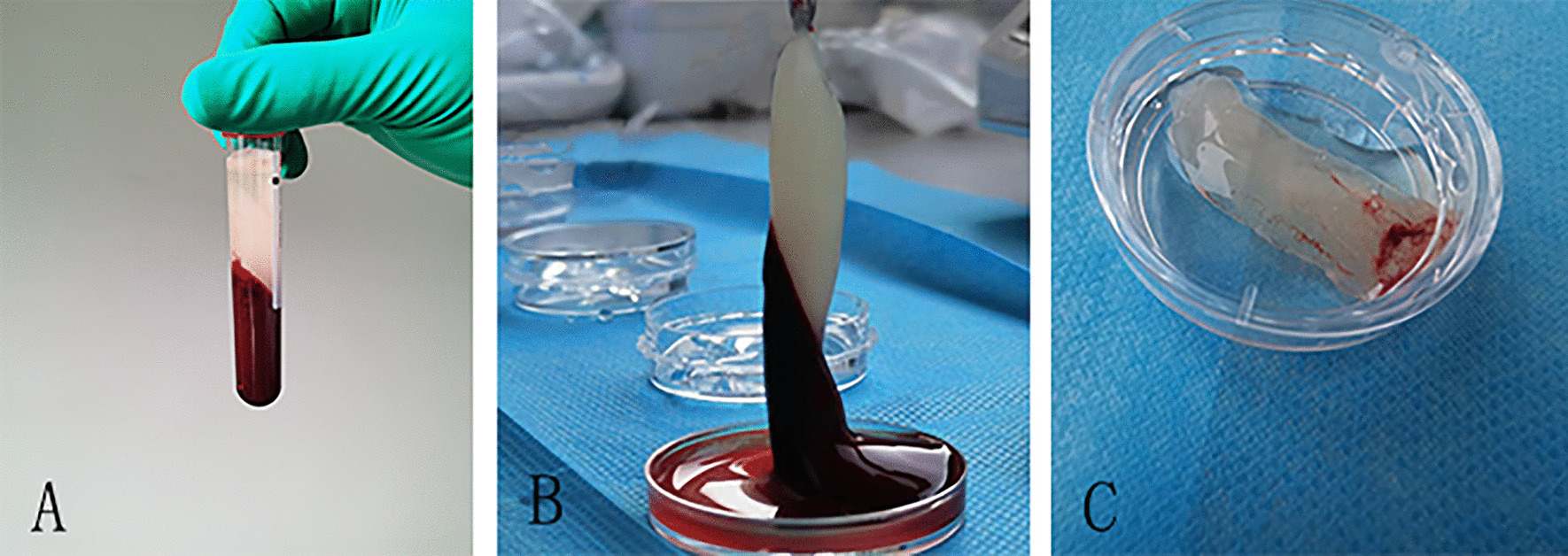
**A** After setting the CGF preparation program, the test tube was divided into three layers. **B** Separate the three layers. **C** Store the CGF gel in sterile saline solution for later use

Nine milliliter venous blood from the ear veins of each rabbit were drawn and collected into sterile vacuum tubes without additive (Greiner BioOne, Kremsmünster). The samples were immediately put into a Medifuge MF200 (Silfradent srl, Forlì, Italy), and centrifugation was carried out according to a preset procedure: acceleration for 30 s, then 2 min at 500RCF, 4 min at 400RCF, 4 min at 500RCF, 3 min at 600RCF, deceleration for 36 s, and stop [15]. This process separated the samples into three layers: a red blood cell (RBC) layer that covered the lower part of the tube, a CGF layer that covered the middle part and a PPP layer that covered the upper part (Fig. 1). The PPP (1 ml) was activated for experimental use with 10% calcium chloride (0.05 ml). The CGF and activated PPP gel were thus collected for experimental use.

Based on a previously described protocol [24], 9 ml venous blood was collected into a conical centrifugal tube (KIRGEN, Shanghai, China) without any anticoagulant. The samples were immediately put into a TD-5Z table centrifuge (Sichuan, China). After centrifuging immediately for 10 min at approximately 1674RCF, the whole blood separated into two layers, the lower layer being the RBC layer, and the upper layer being the PRF layer (Fig. 1).

### Surgical procedure

**Fig. 2:**
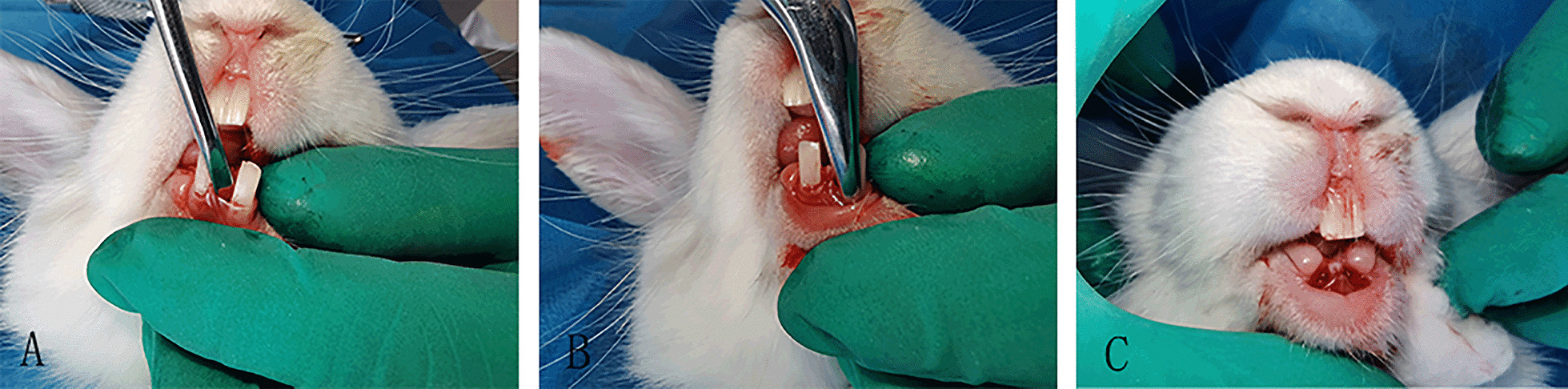
**A** Loosen the lower front teeth with appropriate force. **B** Use the extractor to hold the tooth, shake and pull out. **C** After the extraction of both lower anterior teeth, the extraction wounds were stitched

The animals were randomly and evenly divided into four groups by computer-generated random numbers and kept in sequentially numbered, opaque and closed envelopes. Three groups received PPP, CGF and PRF gel respectively, while the remaining control group did not receive any implant material. They all received an intramuscular injection of penicillin (800,000 units three times daily) for 3 days postoperatively. Intravenous injection of 30 mg/kg sodium pentobarbital (Sigma, St. Louis, MO, USA) through the ear margin was used for general anesthesia. After the anesthetic had taken effect, the gingiva was separated with a periosteal elevator, then the teeth were loosened with the elevator, and after that the bilateral mandibular anterior teeth were extracted. All the above procedures were performed by the same oral and maxillofacial surgeon. Subsequently, the materials were severally implanted into the tooth extraction sockets in the experimental groups (Fig. 2). After that, all the extraction sockets were closed using interrupted sutures of 3/0 absorbable suture (Weihai, Shandong, China). Additionally, all the animals were closely observed for avoiding infection. Thereafter, at 2, 4 and 8 weeks after tooth extraction, three rabbits were randomly selected from each group and euthanized with an overdose of pentobarbital sodium. The bilateral mandible was taken as the specimen for subsequent analysis.

### Radiographic analysis

**Fig. 3:**
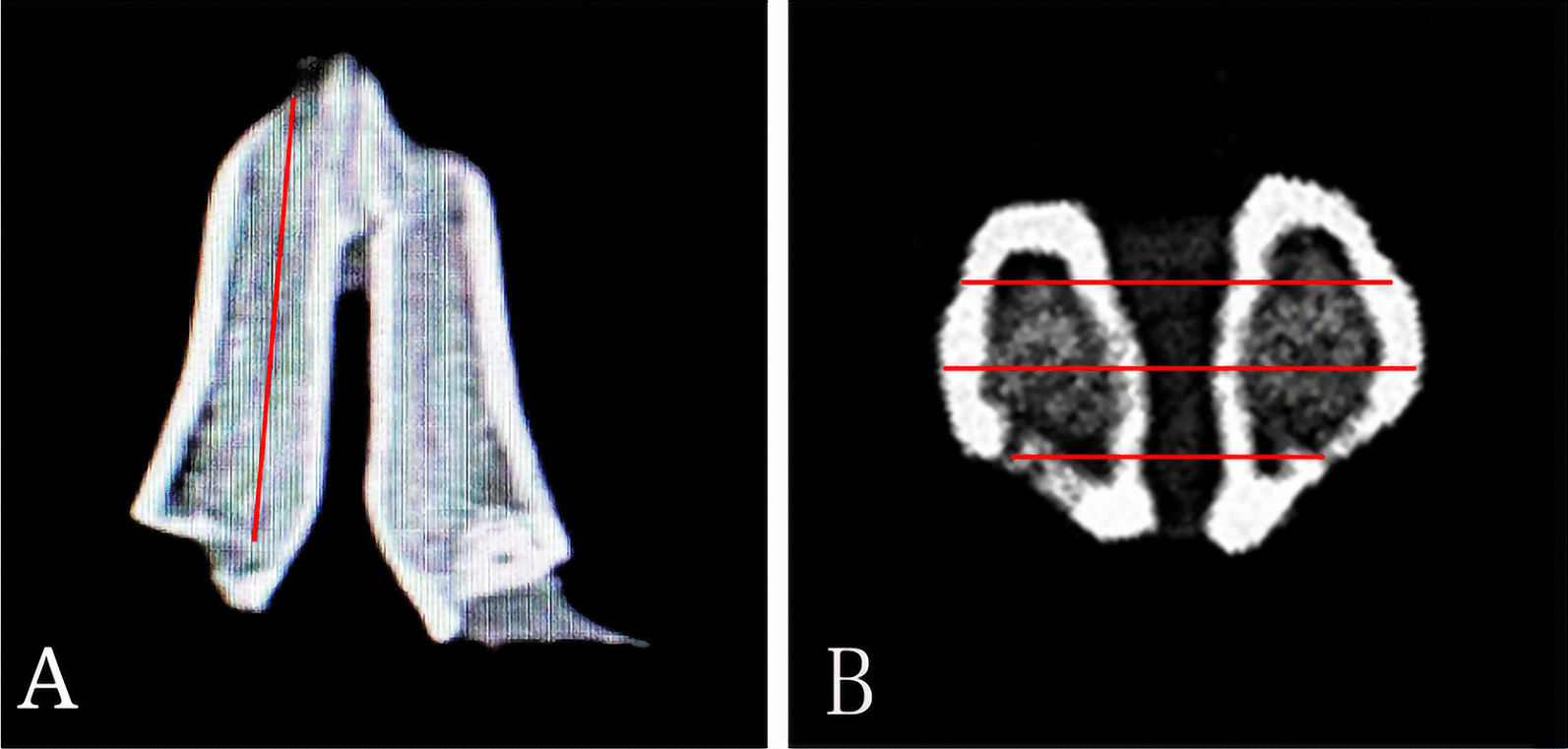
Schematic of the Cone-Beam Computed Tomography (CBCT). **A** Schematic of the measurement of ABH. **B** Schematic of the measurement of ABW

All animals were scanned twice by cone beam computed tomography (CBCT I and CBCT II). CBCT I was obtained after tooth extraction while the animal was still under anesthesia. The anesthetized rabbit was secured to the CBCT chair by a professional radiologist and then adjusted to the appropriate height to complete the CBCT examination. By 2nd, 4th and 8th week, the animal was euthanasia, and CBCT II was obtained after the alveolar bone around the mandibular anterior teeth was immediately removed for scanning. All sectional images were obtained by the same radiologist using a CBCT scanner (i-CAT 17–19, KaVo Group, Shanghai, China), with the following settings: exposure at 5.0 mA and 120 kV for 9.6 s and axial slice thickness 0.2 mm. The results were processed and analyzed by the same radiologist (who was blinded to the group allocation) using image analysis software (CS Imaging Version 7.0.23.0.d, Carestream Health, Rochester, NY, USA). Changes in alveolar bone width (ABW) and alveolar bone height (ABH) were observed. Three sections were selected for each CBCT to measure the height and width respectively, and each section was randomly measured three times. ABW was measured using the method of Chen et al. [25]. Measurements were performed on cross-sectional slices in the apical, median, and coronal third of the socket. ABH was measured using a method described previously by Liu et al. [26] (Fig. 3). Measurement was carried out on three sagittal planes, namely the buccal plane of the extraction socket, the lingual plane, and the middle plane of the first two planes. The changes in ABW and ABH were expressed by the measured value of CBCT before tooth extraction (CBCT I) minus the measured value of CBCT after euthanasia (CBCT II).

### Histological and histomorphometric analysis

After fixing in 10% paraformaldehyde solution for 48 h, the samples were demineralized in 10% EDTA solution (North Tianyi Chemical Reagent Co. Ltd., Tianjin, China) for 5 weeks, washed, dehydrated, and paraffin embedded (Paraplast; Kendall Healthcare, Mansfield, MA, USA), parallel to the long axis of the tooth, comprising a continuous section in the buccal and lingual direction with a section thickness of 5 µm, and then stained with hematoxylin and eosin (H&E). For quantitative analysis, it was performed using the Image J 150e software (National Institutes of Health, Maryland, MD, USA), with a 200× magnification. the percentage of the new bone was calculated with the new bone area/tissue area × 100% (BA/TA). Masson’s trichrome stain was carried out in the same way. The above processes were performed by a histological technician who was blinded to the experimental protocols. An optical microscope (Olympus BX43, Olympus Corporation, Tokyo, Japan) with a magnification of ×200 was used for observation, and a digital camera installed on the microscope was used to obtain images.

### Real-time quantitative polymerase chain reaction (RT-qPCR)

Real-time quantitative polymerase chain reaction (RT-qPCR) was used to detect the expression of two markers of osteogenic genes: a differentiation marker of early osteoblasts—alkaline phosphatase (ALP), and a differentiation marker of late osteoblasts—bone morphogenetic protein-2 (BMP-2). After sacrificing the rabbits, bone tissue was obtained from the tooth extraction sockets and immediately stored in liquid nitrogen and ground in a mortar. A chloroform-free RNA extraction kit (BioTeke, Beijing, China) was used to extract total RNA from the samples. Then, according to the manufacturer's instructions, 1 µg of RNA was reverse transcribed into cDNA, using ReverTra Ace qPCR RT Master Mix (TOYOBO, Japan), and stored at − 20 °C before use. The cDNA was used as the template for real-time quantitative polymerase chain reaction (RT-qPCR). The total volume of the amplification reaction system was 20 µl, including 6 µl primers, 10 µl of SYBR Green PCR Master Mix (TOYOBO, Japan), 1.5 µl cDNA and 2.5 µl ddH_2_O. The primers were purchased from Sangon Biotech Co. (Shanghai, China). The sequences were: 5′-TCCCACTTTGTCTGGAACCG-3′ and 5′-TCCTGTTCAGCTCGTACTGC-3′ for ALP, 5′-AGGAAGCTTTGGGAGACGAC-3′ and 5′-AAGTGGGTCACTTCCACCAC-3′ for BMP, and 5′-GTGGCATCCTGACGCTCAAGTAC-3′ and 5′-AAGCTCGTTGTAGAAGGTGTGGTG-3′ for β-actin.

### Statistical analysis

Data were analyzed using IBM SPSS statistical package 22.0 (IBM Co., Chicago, USA). Categorical variables are presented as the mean ± standard deviation (SD). One-way analysis of variance (ANOVA) with the Student–Newman–Keuls (SNK) comparison test was employed to detect differences among different groups. A significance level of 0.05 was chosen.

## Results

### Clinical results

No accidental deaths occurred. All animals were in good physical condition and had a good diet. No infection or other complications occurred in any of the tooth extraction sockets after surgery. On the 5th day after operation, the tooth extraction wound surfaces of all animals were completely covered by epithelial tissue.

### Radiological analysis

The ABW and ABH gradually increased in all groups over time. However, the experimental groups showed lower amounts of bone resorption.

Two weeks after surgery, the ABW and ABH in each group revealed different degrees of resorption (Table 2). Both ABW and ABH indicated a significantly lower rate of resorption in the PPP group than in the other three groups (*P* < 0.05). The ABW and ABH at 4 and 8 weeks postoperatively are shown in Tables 3 and 4. In short, the CGF group showed the lowest resorption, followed by the PPP and PRF groups, with the highest resorption in the control group. There were no significant differences in ABW between the PRF group and the control group at 4 weeks (*P* > 0.05), or in ABH between the PPP group and CGF group at 8 weeks (*P* > 0.05).

**Table 2:** Resorption of alveolar bone height and width at week 2 according to groups (n = 6)

Group	Resorption of ABW (mm)	Resorption of ABH (mm)
Control	0.21 ± 0.03	0.63 ± 0.06
PPP	0.13 ± 0.02^a,d^	0.44 ± 0.03^a,d^
CGF	0.18 ± 0.04	0.51 ± 0.03^a^
PRF	0.20 ± 0.03^b^	0.56 ± 0.08^b^
*F*	4.000	13.544
*P*-value	< 0.05	< 0.05

Data represent mean ± SD

*ABW* Alveolar bone width, *ABH* alveolar bone height, *PPP* platelet-poor plasma, *CGF* concentrated growth factor, *PRF* platelet-rich fibrin

^a^Statistically significant difference compared to the control group (*P* < 0.05)

^b^Statistically significant difference compared to the PPP group (*P* < 0.05)

^c^Statistically significant difference compared to the CGF group (*P* < 0.05)

^d^Statistically significant difference compared to the PRF group (*P* < 0.05)

**Table 3:** Resorption of alveolar bone height and width at week 4 according to groups (n = 6)

Group	Resorption of ABW (mm)	Resorption of ABH (mm)
Control	0.72 ± 0.08	1.21 ± 0.06
PPP	0.55 ± 0.06^a^	0.91 ± 0.08^a,c,d^
CGF	0.48 ± 0.03^a,d^	0.76 ± 0.08^a,b,d^
PRF	0.63 ± 0.05^c^	1.10 ± 0.05^b,c^
*F*	9.582	26.600
*P*-value	< 0.05	< 0.05

Data represent mean ± SD

*ABW* Alveolar bone width, *ABH* alveolar bone height, *PPP* platelet-poor plasma, *CGF* concentrated growth factor, *PRF* platelet-rich fibrin

^a^Statistically significant difference compared to the control group (*P* < 0.05)

^b^Statistically significant difference compared to the PPP group (*P* < 0.05)

^c^Statistically significant difference compared to the CGF group (*P* < 0.05)

^d^Statistically significant difference compared to the PRF group (*P* < 0.05)

**Table 4:** Resorption of alveolar bone height and width at week 8 according to groups (n = 6)

Group	Resorption of ABW (mm)	Resorption of ABH (mm)
Control	1.53 ± 0.05	1.93 ± 0.04
PPP	0.79 ± 0.05^a,c,d^	1.42 ± 0.04^a,d^
CGF	0.66 ± 0.05^a,b,d^	1.37 ± 0.04^a,d^
PRF	0.98 ± 0.07^a,b,c^	1.68 ± 0.05^a,b,c^
*F*	142.129	101.570
*P*-value	< 0.05	< 0.05

Data represent mean ± SD

*ABW* Alveolar bone width, *ABH* alveolar bone height, *PPP* platelet-poor plasma, *CGF* concentrated growth factor, *PRF* platelet-rich fibrin

^a^Statistically significant difference compared to the control group (*P* < 0.05)

^b^Statistically significant difference compared to the PPP group (*P* < 0.05)

^c^Statistically significant difference compared to the CGF group (*P* < 0.05)

^d^Statistically significant difference compared to the PRF group (*P* < 0.05)

### Histological and histomorphometric analysis

Two weeks after surgery, new bone formation was observed in the tooth extraction sockets in the PPP group, with the new bone extending from the lateral wall to the center. There were abundant osteoblasts and active proliferation. Osteoblasts were arranged in rows around the bone matrix, and some bone trabeculae and mature fibrous tissue could be seen. The new bone trabeculae were thinner in the CGF group and PRF group than in the PPP group, and the arrangement was irregular, with blood vessels growing into the extraction sockets in the PRF group. A large number of inflammatory cells and a small number of fibroblasts were found in the sockets in the control group, and there were few new bones and bone trabeculae.

By the 4th week, each of the experimental groups had formed a larger amount of new bone than the control group. In the CGF group, the new bone further increased and continued to extend towards the center of the sockets, the osteoblasts proliferated actively, the bone trabeculae were more abundant the new bones were connected with each other, and the fibrous connective tissue and inflammatory cells had decreased. New bone formation in the sockets of the PPP group also increased significantly, but there were slightly fewer osteoblasts than in the CGF group, and the bone trabeculae were thinner. The growth of new bone in the PRF group was weaker than that in the PPP group, but there was more neovascularization. In the control group, the small amount of new bone tissue was scattered.

**Fig. 4:**
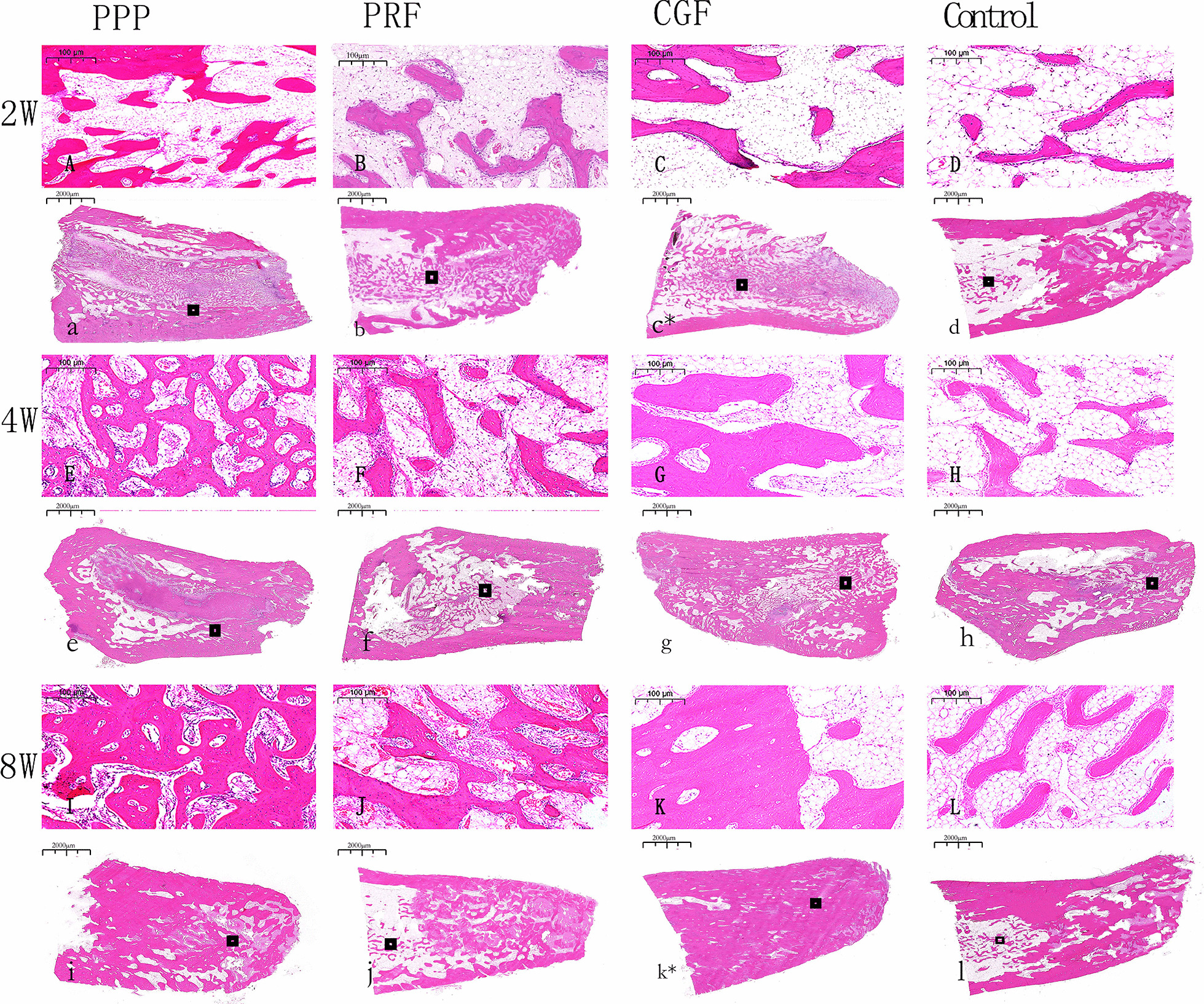
H&E staining of the Tooth extraction at 2, 4 and 8 weeks. n = 6. **A**–**L** Scar bar = 100 µm. **a**–**l** Scar bar = 2000 µm

**Fig. 5:**
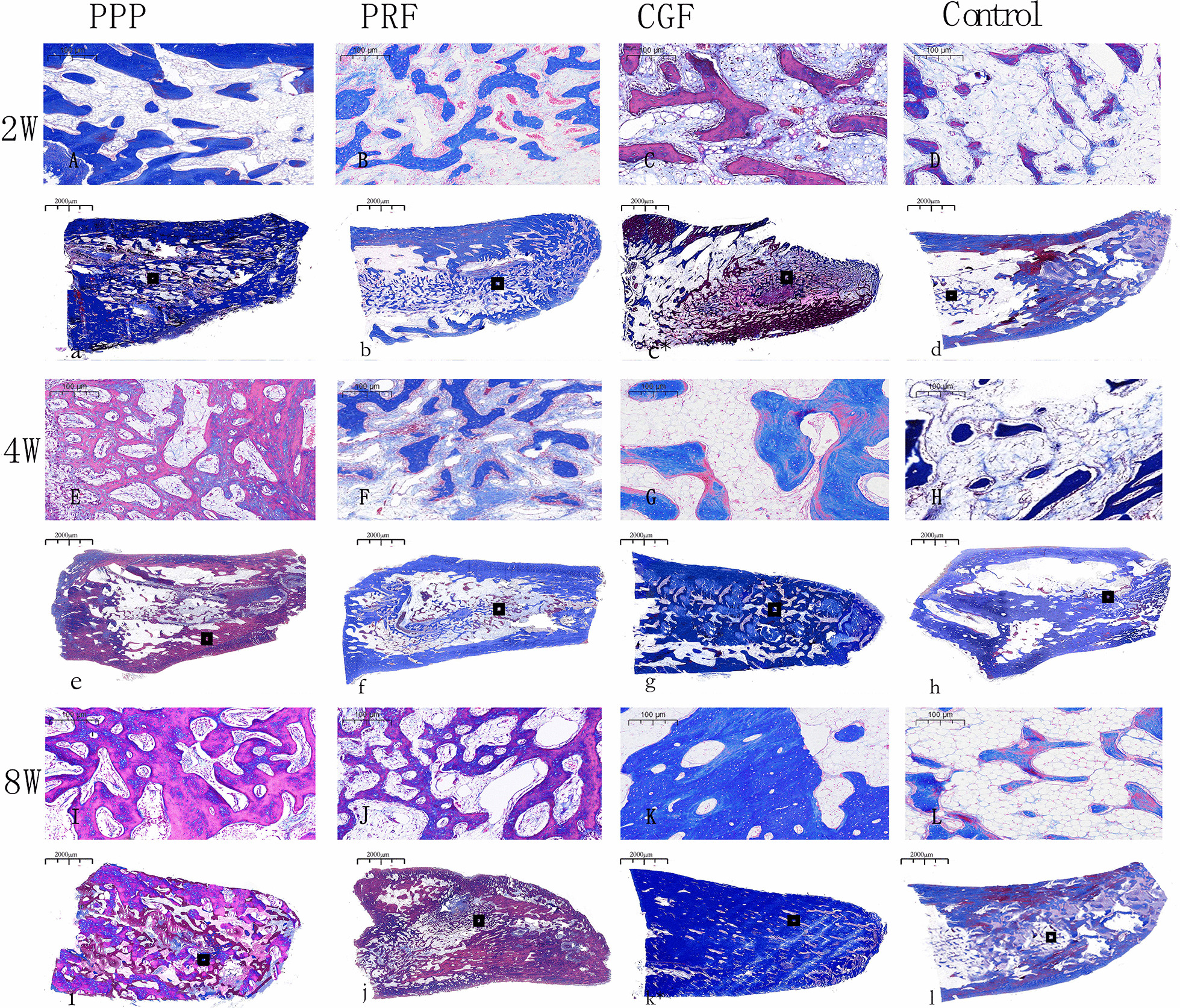
Masson staining of the Tooth extraction at 2, 4 and 8 weeks. n = 6. **A**–**L** Scar bar = 100 µm. **a**–**l** Scar bar = 2000 µm

By the 8th week, new bone had formed in the extraction alveolus in the CGF group, the new bone trabeculae were closely connected and arranged similarly to the normal state, and the trabeculae were thick and calcified, but there was still a small amount of fibrous connective tissue. The new bone in the extraction sockets was thinner in the PPP group followed by the PFR group than that in the CGF group, but the bone tissue of the medial wall of the tooth extraction fossa was more mature and partially fused with the surrounding bone tissue. New bone formation could be seen in the control group, but it was significantly less extensive than in the experimental group, and osteoblasts and blood vessel density were relatively rare (Figs. 4 and 5).

**Fig. 6:**
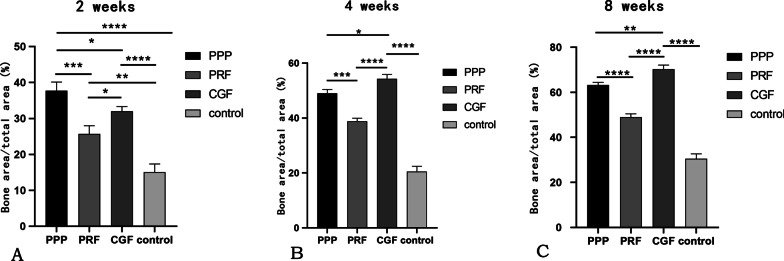
**A** Percentage of new bone by histological study at 2 weeks. *P < 0.05; **P < 0.01; ***P < 0.001; ****P < 0.0001. n = 6. **B** Percentage of new bone by histological study at 4 weeks. *P < 0.05; ***P < 0.001; ****P < 0.0001. n = 6. **C** Percentage of new bone by histological study at 8 weeks. **P < 0.01; ****P < 0.0001. n = 6

The quantitative study of histology was shown in Fig. 6 and also validated the results. By the 2nd week, it can be observed significant differences in all experimental groups when compared to control group (*P* < 0.05), the BA/TA of PPP groups within the four groups was the highest. But over time, the BA/TA of CGF groups was the highest (*P* < 0.05), followed by PPP groups and PRF groups by the 4th and 8th week.

### RT-qPCR analysis

Expression of alkaline phosphatase (ALP): The expression of ALP in the PPP group was the highest at 2 weeks after surgery (*P* < 0.05). At 4 weeks, ALP expression was significantly higher in the CGF group than in the other three groups. By the 8th week, there was no significant difference in ALP activity among the four groups.

**Fig. 7:**
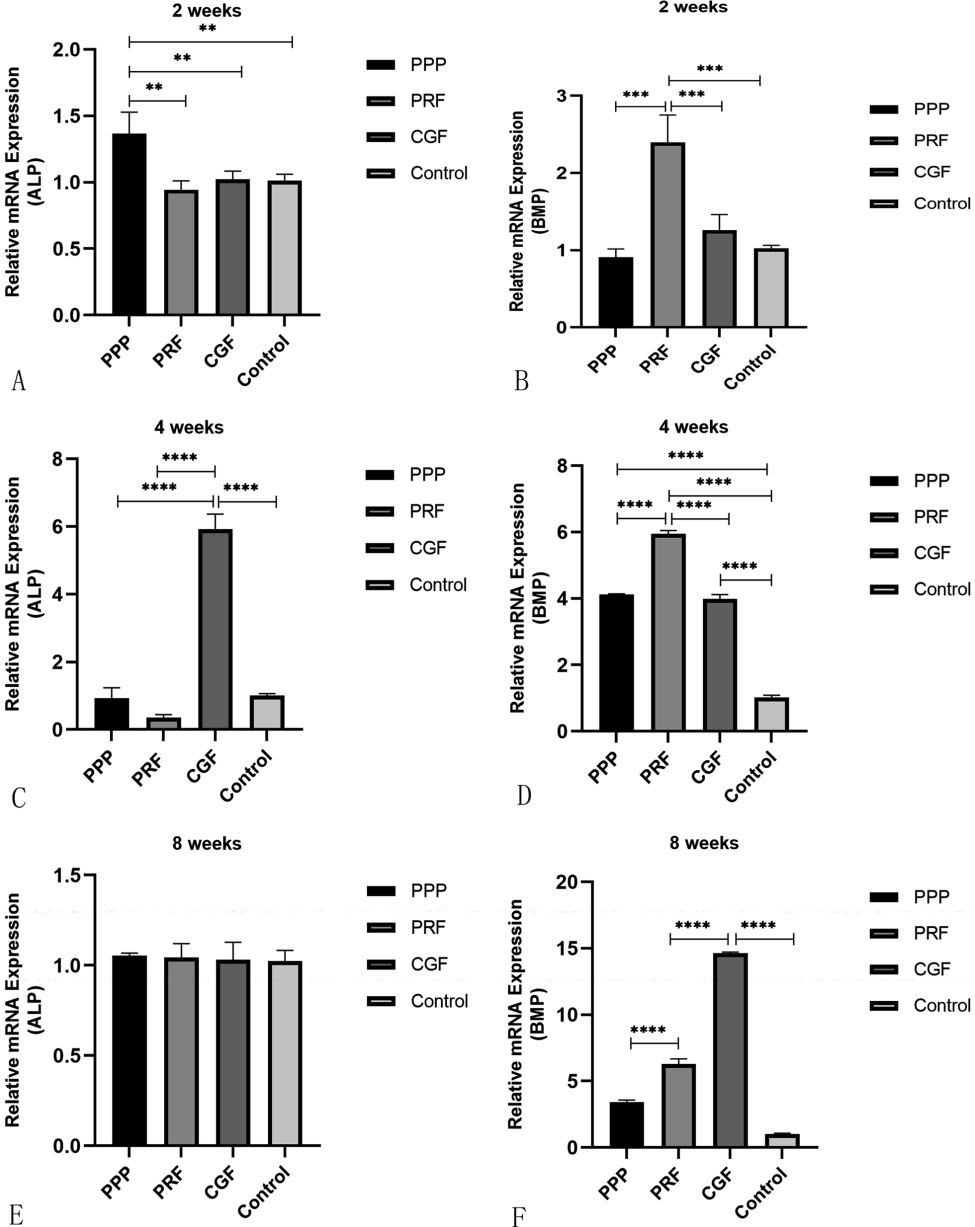
**A** Relative mRNA expression levels of different marker genes (ALP) in bone tissue after tooth extraction filling with PPP, PRF, CGF and control group healed for 2 weeks. ** refers to *P* < 0.01. n = 6. **B** Relative mRNA expression levels of different marker genes (BMP-2) in bone tissue after tooth extraction filling with PPP, PRF, CGF and control group healed for 2 weeks. *** refers to *P* < 0.001. n = 6. **C** Relative mRNA expression levels of different marker genes (ALP) in bone tissue after tooth extraction filling with PPP, PRF, CGF and control group healed for 4 weeks. **** refers to *P* < 0.0001. n = 6. **D** Relative mRNA expression levels of different marker genes (BMP-2) in bone tissue after tooth extraction filling with PPP, PRF, CGF and control group healed for 4 weeks. **** refers to *P* < 0.0001. n = 6. **E** Relative mRNA expression levels of different marker genes (ALP) in bone tissue after tooth extraction filling with PPP, PRF, CGF and control group healed for 8 weeks. n = 6. **F** Relative mRNA expression levels of different marker genes (BMP-2) in bone tissue after tooth extraction filling with PPP, PRF, CGF and control group healed for 8 weeks. **** refers to *P* < 0.0001. n = 6

Expression of bone morphogenetic protein-2 (BMP-2): BMP expression was significantly higher in the PRF group than in the other groups at 2 weeks. The PRF group still had the highest expression at 4 weeks, and at the same time, the expression of BMP in the PPP group and CGF group was gradually increasing. By the 8th week, with the development of bone remodeling, BMP expression was the highest in the CGF group, followed by the PRF group and PPP group, which all showed higher expression than the control group (*P* < 0.05) (Fig. 7).

## Discussion

A series of events occur during the healing of tooth extraction sockets, including (1) blood clot formation, (2) fibroblast infiltration and vascular endothelial cell proliferation, (3) connective tissue hyperplasia, (4) fibrous bone formation, and (5) mature bone tissue establishment. The natural healing process of the entire extraction sockets will be affected by systemic biological factors such as age, gender, and hormone levels, especially sex hormones [27]. At the same time, local microvascular formation also plays a very important and critical role in the growth and development of the woven bone. In addition, the healing process involves interactions between various cells and growth factors [28]. Therefore, improving the microenvironment of extracted sockets by providing the growth factors required for healing can improve the healing conditions and promote the healing of extracted sockets and induce bone tissue regeneration.

PRF and CGF are platelet concentrates containing a large number of growth factors [29], including transforming growth factor (TGF-β), platelet-derived growth factor (PDGF), vascular endothelial growth factor (VEGF), and basic fibroblast growth factor (BFGF). These growth factors participate in events such as osteoblastic movement, proliferation and differentiation [30], so as to regulate the activity of osteoblasts and promote bone regeneration.

PPP is the upper component of autologous plasma products. Compared with PRF and CGF, it contains fewer platelets, but is rich in plasma growth factor and fibrinogen. Chen et al. [31] showed that fibrinogen may have a more significant effect on bone regeneration, suggesting that PPP has the potential to promote bone regeneration in the early stages of healing. Here, the questions arose. Can three autologous blood products be used for preservation of alveolar bone after tooth extraction? How should they be selected in clinical practice? For this purpose, in this study, PPP, CGF and PRF were applied to rabbit extraction sockets to observe and compare their effects on wound healing and alveolar bone resorption in order to identify more appropriate options for filling extraction sockets and to develop new clinical treatment methods.

The radiographic results showed that the resorption of ABW and ABH in the experimental groups were lower than the control group at all observation points, suggesting that autologous plasma products can effectively promote tooth extraction wound healing. And the study of Srinivas et al. supported the results [32]. The histological and histomorphometric analyses revealed large areas of new alveolar bone in all three experimental groups at all time points, except for the control group, where only a small amount of new alveolar bone was generated, similar to the findings of other scholars. For example, Hatakeyama et al. [33] found that in their vivo study of rat calvarial bone defects, the bone defects were almost filled with bone tissue 8 weeks after surgery and treatment with CGF and PPP gel, while a few control (untreated) defects were still apparent at 8 weeks. Similarly, Kim et al. [8] observed that in their comparative study on rabbit-skull defect healing, the PRP, PRF and CGF groups had all formed a higher amount of new bone than the control group by the 6th week after surgery. In addition, the three autologous concentrates could not be histologically observed at 2 weeks after surgery, so it was speculated that they might be completely degraded within 2 weeks after being put into the tooth sockets. The study of Isobe et al. [34] and Wang [35] supported the observation.

During the growth of osteoblasts, the expression of specific genes varies at different stages [36]. The peak expression of genes reflects the developmental sequence of osteocyte differentiation, which can be divided into three main stages: proliferation, extracellular matrix maturation and mineralization. Some scholars have proposed that the regulation of genes in this developmental sequence depends on the maturation of osteoblasts [37]. ALP activity is closely related to bone growth and remodeling, and its expression can reflect the early differentiation of osteoblasts and the maturity of bone tissue [38]. BMP-2 promotes the maturation and function of osteoblasts and bone remodeling by inducing the differentiation of mesenchymal bone progenitor cells [39]. It is a representative osteogenic product in the late stage of osteogenic differentiation. Our results showed that the expression peak of BMP lagged behind that of ALP during alveolar bone healing in all groups, which was similar to the results of other scholars, Lu et al. And these studies showed that the expression of ALP was biphasic: ALP was expressed at a high level in the early stage of osteogenesis, but with the development of bone maturation and matrix mineralization, it reduced gradually, and the late marker of osteoblast differentiation increased [40]. In addition, the qRT-PCR results of this study also showed that the expression of ALP was the highest in PPP group at 2 weeks, while in CGF group at 4 weeks. This indicated that the effect of PPP was more obvious in the early stage of bone healing, while the effect of CGF was slower, but CGF was more effective than the other two in the whole observation period. The reasons for this phenomenon may be credit to their different biological structures. The structure of CGF fiber is denser, and its growth factor has the characteristic of slow release [41]. At the 8th week, with the gradual maturation of the bone in all groups, the expression of ALP in each group was gradually down-regulated and kept at a relatively stable level, and there was no significant difference in the expression level of ALP among the four groups. However, according to Isobe et al., the three autologous plasma products released a large number of growth factors as they gradually degraded [34], and this may be the reason why we could still observe that the experimental groups showed better healing effect than the control group at a later stage. Furthermore, the osteogenic signaling pathways might be activated in different degrees in the three experimental groups, resulting in differences in the progress of bone healing in each experimental group on the whole. The experimental group accelerated the healing process to an overall extent and the expression of BMP showed that all experimental groups entered the bone mineralization phase earlier than the control group and showed higher mineralization levels and better healing effect. This was consistent with the results of histological analysis in this experiment: the PPP group showed more active bone growth at the 2nd week, while the new bone growth and differentiation level of the CGF group was better than that of the other three groups after the 4th and 8th week.

Overall, CGF and PPP were slightly more effective than PRF at promoting extraction socket healing during the time points observed in our study. The reason for this may be that the rich fibrin fibers and fibrinogen in CGF and PPP play an important role in promoting the healing of tooth extraction sockets. Moreover, Hatakeyama et al. [33] showed that significantly more fibrinogen was contained within PPP than in PRF, and Isobe et al. [34] found that CGF gels contained thicker fibrin fibers than PRF gels based on scanning electron microscopic examination. Both findings support our results and conjecture.

In addition, it is found that the effect of PPP was slightly superior to PRF and CGF in early healing. This may be because CGF and PRF, which contain a large number of platelets, are not only reservoirs of growth factors but also immune nodes containing a large number of inflammatory mediators. Inflammatory factors such as α -granules released after their activation may limit the differentiation of osteoblast-related cells during early healing process [42, 43]. Moreover, CGF significantly outperformed PPP and PRF in promoting osteogenesis at later stage. The reason for this may be that the fibrin network of insoluble fibrin provides a scaffold for the cells and serves as a substrate for the continuous release of growth factors, and the cells are exposed to fibrin molecules exhibiting three-dimensional cell–cell interactions [44], allowing the growth factors to continuously act on the extraction sockets, promoting osteoblast proliferation and differentiation as well as reducing the resorption of the alveolar bone. Compared with PRF, CGF not only has a higher fibrinogen, but also has a more stable fibrinogen network, which can prevent plasma mediated degradation [45], which may be due to the special centrifugal process of CGF. CGF special centrifuges are equipped with an oxygenation mechanism that prevents temperature increases and helps maintain the growth of cells contained in the fibrin matrix. In addition, the fibrin network in CGF provides a three-dimensional cell–cell interaction that allows growth factors to continue to act on tooth extraction. In addition, studies have shown that osteoblast differentiation is regulated by soluble GF such as TGF-β1, PDGF-AB and IGF-1. CGF contains a variety of GF and proteins, while PPP only contains IGF-1 in serum, which may be the reason why CGF is superior to PPP in osteogenic induction [46]. Therefore, since PPP was more advantageous in promoting early osteogenesis after extraction, in clinical situations where immediate or early implantation after extraction is required, we can choose PPP. And in cases where delayed implantation is required, CGF might be chosen for filling the extraction sockets.

One limitation of the study is that it failed to further explore the molecular biological mechanisms underlying the effect of PRF, CGF and PPP on the healing of tooth extraction sockets. However, real time PCR were preliminarily used to observed the expression level of osteogenic genes in different groups. In addition, platelet concentrates could be used in combination with other material to optimize their effects. Further clinical study would be conducted to verify and supplement the results of this study.

## Conclusions

PPP, PRF and CGF can promote the healing of tooth extraction sockets, promote new bone formation, reduce bone resorption, and improve the expression of osteogenesis-related genes. Yet considering their long-term effects, CGF shows greater benefits in osteogenesis, resulting in efficient bone induction and tissue regeneration. Since the components of PPP, CGF and PRF are all derived from autologous blood without immunogenicity, their preparation is simple. Furthermore, they have good biocompatibility and appropriate biodegradability when they are implanted into the tooth extraction sockets.

## Data Availability

The datasets generated and/or analysed during the current study are not publicly available due to the ongoing related further research projects but are available from the corresponding author on reasonable request.

## References

[1] Barone A, Ricci M, Tonelli P, Santini S, Covani U (2013). Tissue changes of extraction sockets in humans: a comparison of spontaneous healing vs. ridge preservation with secondary soft tissue healing. Clin Oral Implants Res..

[2] Discepoli N, Vignoletti F, Laino L, de Sanctis M, Muñoz F, Sanz M (2013). Early healing of the alveolar process after tooth extraction: an experimental study in the beagle dog. J Clin Periodontol.

[3] Stumbras A, Kuliesius P, Januzis G, Juodzbalys G (2019). Alveolar ridge preservation after tooth extraction using different bone graft materials and autologous platelet concentrates: a systematic review. J Oral Maxillofac Res..

[4] Gholami GA, Najafi B, Mashhadiabbas F, Goetz W, Najafi S (2012). Clinical, histologic and histomorphometric evaluation of socket preservation using a synthetic nanocrystalline hydroxyapatite in comparison with a bovine xenograft: a randomized clinical trial. Clin Oral Implants Res.

[5] Adel-Khattab D, Afifi NS, Abu El Sadat SM, Aboul-Fotouh MN, Tarek K, Horowitz RA (2020). Bone regeneration and graft material resorption in extraction sockets grafted with bioactive silica-calcium phosphate composite (SCPC) versus non-grafted sockets: clinical, radiographic, and histological findings. J Periodontal Implant Sci..

[6] Chen ZY, Gao S, Zhang YW, Zhou RB, Zhou F (2021). Antibacterial biomaterials in bone tissue engineering. J Mater Chem B.

[7] Zhang Z, Li X, Zhao J, Jia W, Wang Z (2019). Effect of autogenous growth factors released from platelet concentrates on the osteogenic differentiation of periodontal ligament fibroblasts: a comparative study. PeerJ..

[8] Kim TH, Kim SH, Sándor GK, Kim YD (2014). Comparison of platelet-rich plasma (PRP), platelet-rich fibrin (PRF), and concentrated growth factor (CGF) in rabbit-skull defect healing. Arch Oral Biol.

[9] Li F, Jiang P, Pan J, Liu C, Zheng L (2019). (2019) Synergistic application of platelet-rich fibrin and 1% alendronate in periodontal bone regeneration: a meta-analysis. BioMed Res Int.

[10] Castro AB, Meschi N, Temmerman A, Pinto N, Lambrechts P, Teughels W (2017). Regenerative potential of leucocyte- and platelet-rich fibrin. Part A: intra-bony defects, furcation defects and periodontal plastic surgery. A systematic review and meta-analysis. J Clin Periodontol..

[11] Marchetti E, Mancini L, Bernardi S, Bianchi S, Cristiano L, Torge D, Marzo G, Macchiarelli G (2020). Evaluation of different autologous platelet concentrate biomaterials: morphological and biological comparisons and considerations. Materials (Basel, Switzerland).

[12] Tarallo F, Mancini L, Pitzurra L, Bizzarro S, Tepedino M, Marchetti E (2020). Use of platelet-rich fibrin in the treatment of grade 2 furcation defects: systematic review and meta-analysis. J Clin Med.

[13] Fernández-Medina T, Vaquette C, Ivanovski S (2019). Systematic comparison of the effect of four clinical-grade platelet rich hemoderivatives on osteoblast behaviour. Int J Mol Sci.

[14] Sacco L. International academy of implant prosthesis and osteoconnection. In: Lecture. 2006.

[15] Rodella LF, Favero G, Boninsegna R, Buffoli B, Labanca M, Scarì G (2011). Growth factors, CD34 positive cells, and fibrin network analysis in concentrated growth factors fraction. Microsc Res Tech.

[16] Talaat WM, Ghoneim MM, Salah O, Adly OA (2018). Autologous bone marrow concentrates and concentrated growth factors accelerate bone regeneration after enucleation of mandibular pathologic lesions. J Craniofac Surg.

[17] Xu Y, Qiu J, Sun Q, Yan S, Wang W, Yang P (2019). One-year results evaluating the effects of concentrated growth factors on the healing of intrabony defects treated with or without bone substitute in chronic periodontitis. Med Sci Monit.

[18] Creeper F, Lichanska AM, Marshall RI, Seymour GJ, Ivanovski S (2009). The effect of platelet-rich plasma on osteoblast and periodontal ligament cell migration, proliferation and differentiation. J Periodontal Res.

[19] Song D, Huang Y, Van Dessel J, Shujaat S, Orhan K, Vangansewinkel T (2019). Effect of platelet-rich and platelet-poor plasma on peri-implant innervation in dog mandibles. Int J Implant Dent.

[20] Chellini F, Tani A, Zecchi-Orlandini S, Sassoli C (2019). Influence of platelet-rich and platelet-poor plasma on endogenous mechanisms of skeletal muscle repair/regeneration. Int J Mol Sci.

[21] Hamdan AA, Loty S, Isaac J, Bouchard P, Berdal A, Sautier JM (2009). Platelet-poor plasma stimulates the proliferation but inhibits the differentiation of rat osteoblastic cells in vitro. Clin Oral Implants Res.

[22] Kilkenny C, Browne W, Cuthill IC, Emerson M, Altman DG; NC3Rs Reporting Guidelines Working Group. Animal research: reporting in vivo experiments: the ARRIVE guidelines. Br J Pharmacol. 2010;160:1577–1579. 10.1111/j.1476-5381.2010.00872.x PMC2936830 20649561

[23] Directive of No 2010/63/EU of the European parliament and of the council of 22 September on the protection of animals used for scientific purposes. Chin J Zoonoses. 2010;39(4):741–750.

[24] Dohan DM, Choukroun J, Diss A, Dohan SL, Dohan AJ, Mouhyi J, Gogly B (2006). Platelet-rich fibrin (PRF): a second-generation platelet concentrate. Part I: technological concepts and evolution. Oral Surg Oral Med Oral Pathol Oral Radiol Endod..

[25] Chen J, He Y, Shan C, Pan Q, Li M, Xia D (2015). Topical combined application of dexamethasone, vitamin C, and β-sodium glycerophosphate for healing the extraction socket in rabbits. Int J Oral Maxillofac Surg.

[26] Liu X, Zhang X, Tian X, Chen J, He Y (2021). Effect of osteogenic inducer sustained release system on bone remodeling after tooth extraction. Acts Universitatis Medicinalis Anhui.

[27] Shimizu M, Furuya R, Kawawa T (2000). Bone wound healing after maxillary molar extraction in ovariectomized aged rats: quantitative backscattered electron image analysis. Anat Rec.

[28] Lee JH, Kim JW, Lee JH, Chung KJ, Kim TG, Kim YH, Kim KJ (2018). Wound healing effects of paste type acellular dermal matrix subcutaneous injection. Arch Plast Surg.

[29] Lee HM, Shen EC, Shen JT, Fu E, Chiu HC, Hsia YJ (2020). Tensile strength, growth factor content and proliferation activities for two platelet concentrates of platelet-rich fibrin and concentrated growth factor. J Dent Sci.

[30] Li H, Sun S, Liu H, Chen H, Rong X, Lou J (2016). Use of a biological reactor and platelet-rich plasma for the construction of tissue-engineered bone to repair articular cartilage defects. Exp Ther Med.

[31] Chen TL, Liang XJ, Zhang XH (2019). Do the fibrin scaffold and growth factors in platelet-rich fibrin play the most vital roles in bone regeneration? A Critical Comment. J Craniofac Surg.

[32] Srinivas B, Das P, Rana MM, Qureshi AQ, Vaidya KC, Ahmed Raziuddin SJ (2018). Wound healing and bone regeneration in postextraction sockets with and without platelet-rich fibrin. Ann Maxillofac Surg.

[33] Hatakeyama I, Marukawa E, Takahashi Y, Omura K (2014). Effects of platelet-poor plasma, platelet-rich plasma, and platelet-rich fibrin on healing of extraction sockets with buccal dehiscence in dogs. Tissue Eng Part A.

[34] Isobe K, Watanebe T, Kawabata H, Kitamura Y, Okudera T, Okudera H (2017). Mechanical and degradation properties of advanced platelet-rich fibrin (A-PRF), concentrated growth factors (CGF), and platelet-poor plasma-derived fibrin (PPTF). Int J Implant Dent.

[35] Wang J, Wang L, Zhou Z, Lai H, Xu P, Liao L (2016). Biodegradable polymer membranes applied in guided bone/tissue regeneration: a review. Polymers.

[36] Yoon JY, Kim TS, Ahn JH, Yoon JU, Kim HJ, Kim EJ (2019). Remifentanil promotes osteoblastogenesis by upregulating Runx2/osterix expression in preosteoblastic C2C12 cells. J Dent Anesth Pain Med.

[37] Lian JB, Stein GS (1992). Concepts of osteoblast growth and differentiation: basis for modulation of bone cell development and tissue formation. Crit Rev Oral Biol Med.

[38] Tang H, Kankala RK, Wang S, Chen A (2019). Supercritical fluid-assisted controllable fabrication of open and highly interconnected porous scaffolds for bone tissue engineering. Sci China Life Sci.

[39] Mai Z, Peng Z, Wu S, Zhang J, Chen L, Liang H (2013). Single bout short duration fluid shear stress induces osteogenic differentiation of MC3T3-E1 cells via integrin β1 and BMP2 signaling cross-talk. PLOS ONE.

[40] Lu Y, Li L, Zhu Y, Wang X, Li M, Lin Z (2018). Multifunctional copper-containing carboxymethyl chitosan/alginate scaffolds for eradicating clinical bacterial infection and promoting bone formation. ACS Appl Mater Interfaces.

[41] Tian S, Wang J, Dong F, Du N, Li W, Song P (2019). Concentrated growth factor promotes dental pulp cells proliferation and mineralization and facilitates recovery of dental pulp tissue. Med Sci Monit.

[42] Hong S, Chen W, Jiang B (2018). A comparative evaluation of concentrated growth factor and platelet-rich fibrin on the proliferation, migration, and differentiation of human stem cells of the apical papilla. J Endod.

[43] Golebiewska EM, Poole AW (2015). Platelet secretion: From haemostasis to wound healing and beyond. Blood Rev.

[44] Kawase T, Okuda K, Saito Y, Yoshie H (2005). In vitro evidence that the biological effects of platelet-rich plasma on periodontal ligament cells is not mediated solely by constituent transforming-growth factor-beta or platelet-derived growth factor. J Periodontol.

[45] Park HC, Kim SG, Oh JS, You JS, Kim JS, Lim SC (2016). Early bone formation at a femur defect using CGF and PRF grafts in adult dogs: a comparative study. Implant Dent.

[46] Takeda Y, Katsutoshi K, Matsuzaka K, Inoue T (2015). The effect of concentrated growth factor on rat bone marrow cells in vitro and on calvarial bone healing in vivo. Int J Oral Maxillofac Implants.

